# Reasons for transferral to emergency departments of terminally ill patients - a French descriptive and retrospective study

**DOI:** 10.1186/s12904-016-0155-y

**Published:** 2016-10-21

**Authors:** Pierre Cornillon, Sébastien Loiseau, Bruno Aublet-Cuvelier, Virginie Guastella

**Affiliations:** 1 Department of Palliative Care, Centre hospitalier Universitaire, Hopital Nord, 63000 Clermont-Ferrand, France; 2 Emergency Department, Centre Hospitalier d’Issoire, Issoire, France

**Keywords:** Palliative care, Hospital-at-home, Emergency departments, Avoidable presentations

## Abstract

**Background:**

Patients under palliative care and in hospital-at-home services are frequently transferred to emergency departments.

We set out to identify the reasons for these presentations to determine the proportion that might be avoidable.

**Methods:**

We conducted a retrospective study by assessment of patient files.

We studied admissions to four emergency departments in an area of France (Puy-de-Dôme) between September 2011 and August 2013. Reasons for transfer and diagnostic conclusion by emergency doctors were noted. We collected date of admission, time spent, investigations and treatments performed and patients’ outcomes after the medical conclusions. We also determined whether patients called the hospital-at-home service before going to the emergency department. From these data we discerned potentially avoidable and unavoidable consultations.

**Results:**

We identified 52 transfers of patients from home to emergency departments. The most frequent reasons were: generalized weakness (11 cases), social isolation (8 cases) and end of life (7 cases). For 58 % of presentations, the investigations and treatments performed did not require presentation to an emergency department; 34 % of patients returned home after the visit, 41 % remained for simple observation and 20 % remained to receive special care. Two patients died in the emergency department. In 86 % of cases, presentations occurred when primary care was less readily available, and patients called home care services in only 42 % of cases before going to emergency departments.

**Conclusions:**

Half of the transfers to emergency departments were potentially avoidable for terminally ill patients in home care. To reduce this proportion we need to promote access to primary care, educate patients in hospital-at-home service and train care-givers and doctors in palliative medicine.

**Electronic supplementary material:**

The online version of this article (doi:10.1186/s12904-016-0155-y) contains supplementary material, which is available to authorized users.

## Key statements

1. What is already known about the topic:
  In international literature several studies describe avoidable admissions to emergency departments for terminally ill patients.
  No such studies have been conducted in France.
  Many reasons are advanced for these avoidable admissions. However, we found no prospective study that proposes concrete solutions. None of the solutions described are evidence-based.
2. What this paper adds:
  This study looked at four local French emergency departments to assess whether the French system leads to avoidable admissions for terminally ill patients, and if so what the reasons are for this dysfunction.
  This work is preliminary to a prospective study whose aim will be to suggest concrete solutions to avoid inappropriate ED transferral. Prior to prospective work, we first needed to evaluate the current situation.
3. Implications for practice, theory or policy:
  Assessing the causes of avoidable admissions will help us find suitable ways to reduce their number: promote a round-a-clock medical intervention and improve education of patients and their families to be sure they call their referent doctors before consulting in Emergency Department.
  Training of medical staff to help them take adapted decisions.
  Problems in the French system are probably similar to those elsewhere. Hence adjustments we propose for France could apply in other countries.

## Background

Many studies have shown that people prefer to be cared for and die at home [1, 2]. A report of the Health Foundation states that under the right circumstances, community-based services can be an effective alternative to hospital treatment [3]. This report also found that patients expressed greater satisfaction with treatment-at-home regimes than with hospital inpatient care. Currently there is no evidence that hospital-at-home (HAH) results are poorer than those of acute hospital wards. A Cochrane review reported a lack of evidence for superiority of HAH [4]. Hospital-at-home organization differs across countries. Some services are based at the local hospital and employ many physicians and nurses. Others use independent contractors to provide equipment [3].

French people today say they would mostly prefer to die at home [3]. Studies show that 50–80 % of the French wish to die at home, but only 30 % actually do so [3, 4]. In France, many terminally ill patients who prefer to be hospitalized at home can receive care through special services [5]. France is developing HAH services for terminally ill patients [6]. This shortens time spent in hospital, in line with patients’ wishes [7, 8]. Home hospitalization services are broadly diversified. Some have mostly a coordination function, while others have nurses visiting patients frequently. Generally they use independent nurses or nursing assistants to deliver much of the care. Pharmaceuticals can be supplied by the pharmacy of the HAH service (or at the hospital) or by a pharmacist outside the hospital [9].

This specific feature requires perfect coordination between the different actors in a patient’s care path to avoid unneeded care, inappropriate investigations or superfluous hospital admissions. Family physicians are called upon to play many different complex roles to prevent hospital admission at end of life [10].

In this context many patients in palliative care can be faced with emergency problems at home. They often consult emergency departments (EDs) for various problems [11–14]. These presentations are often uncomfortable for both patients and families [15] because wards are ill-adapted and EDs are overcrowded, so that care-givers cannot give enough time. The main focus of the ED is to manage persons with acute illness or trauma, but persons with chronic and advanced disease, and those facing end-of-life emergencies, are presenting at EDs across the country in increasing numbers [16]. A study conducted in Belgium and France found that death in emergency departments mainly concerned elderly patients with multiple chronic diseases, and was frequently preceded by a decision to withdraw and/or withhold life support [17]. The time spent in ED is rarely valued by either patients or families. These patients have often a wide variety of symptoms and they need extended support, difficult to achieve in an ED. Hence they are at high risk of suffering from serious conditions [18]. A French study concluded that palliative care is administered to only about half of the patients who die in EDs [19]. Early planned hospitalization in a medical department or home intervention would be better for patients, families and care-givers.

Care can also be difficult for nurses and doctors in EDs because their ways of working are based on principles that are totally different from those of palliative care [20]. Care-givers can also experience difficulties approaching these complex situations, for which they have little training [21]. The complexity of these situations needs specific knowledge of palliative principles and time to adapt them for each patient [22].

French and other studies conclude that ED presentations for this category of patients are not essential [23, 24]. In the international literature they are called “potentially avoidable presentations "(PAPs) [25]. The causes of PAPs are not established in France, especially not for home-hospitalized patients. Defining the medical and social reasons that lead to ED presentations would help avoid some of them [26] by forward planning or transferring them directly to a more suitable medical ward.

### Aim

This study aimed to determine the reasons for presentation to emergency departments of home-hospitalized terminally ill patients to determine whether or not these presentations were potentially avoidable.

### Design

We conducted a retrospective and multicentre chart audit in the Puy-de-Dôme area of France lasting 24 months between 1 September 2011 and 31 August 2013. It included four EDs and three hospital-at-home services.

The Puy-de-Dôme is a department in the middle of France with about six hundred thousand inhabitants. Its population is divided in two, half rural and half urban (see map).

## Methods

We included all the patients of any age who were in a clearly defined palliative situation, who were home hospitalized, and who presented to ED during the study period. In this context the informed consent of patients was impossible because most of them were dead at the start of the study. Of the four EDs, the biggest one is in the University Hospital and supports about 150 patients per day. The others belong to small peripheral hospitals and support about 40 patients each per day.

The palliative situation was pronounced by a multidisciplinary team during a multidisciplinary meeting in the patients’ reference facility. The exclusive palliative care decision was essentially based on global weakness and physical incapacity to support heavy treatments. The palliative care status was officially identified and patients were referred to the HAH service with this designation.

Selected patients were followed at two HAH services. The 2 HAH who participated to the study support about 150 patients each per year with 80 % of their patients clearly defined as needing palliative care. The third one declined to participate in the study, but its palliative activity was negligible compared to the others.

We selected patients using the HAHs’ presentations database, in which patient transfers are recorded by the HAH doctors. We accessed these with the agreement of the HAHs. We provided written oath of professional confidentiality. We then added data from ED medical records.

For each of these presentations we collected qualitative data: reasons for the presentation and emergency doctor’s diagnosis; the location of the visit; the examinations or medical interventions performed during the visit. We also studied the patient’s outcome after the visit.

Quantitative data were also collected: the age of the patient calculated from date of birth, time spent in ED calculated from administrative files, distance and time between patient’s home and closest ED: the distance was assessed by the software Mappy®.

We defined an avoidable presentation as one with no examination or medical intervention, or with simple examinations: radiology, electrocardiogram and biological analyses (**avoidable group**). We judged these as avoidable because they can be done easily outside of hospital. It would have allowed an adapted and programmed answer without spending time in an emergency department. We defined a presentation as unavoidable if the patient was examined by scanner/MRI, echography or received a specialist intervention (**unavoidable group**).

Data were collected by the same investigator throughout the study to avoid bias.

We also report what happened to patients after the visit: returned home, hospitalized in a medical/surgical ward, or hospitalized briefly in a post-emergency ward. We noted the reason for the hospitalization: care or simple observation.

We determined the date and time of presentation: weekday or weekend, vacation times, public holidays, day or night.

Lastly we checked whether the HAH service had been called before patients consulted an ED.

## Results

In all 48 patients from two HAH services made 52 ED visits, 4 making two visits each.

Compared with the overall number of patients in a palliative situation who were home-hospitalized, we estimated that 21 % of home-hospitalized patients visited an ED. Of these 60 % were men, with an average age of 69.5 years; 43 patients suffered from an oncological illness, five had neurological disorders, and four had advanced organ failure. Prescriptions were made in anticipation of potential symptoms at home. None of the patients had an advance care directive in place. Patients’ characteristics are given in Table 1.

**Table 1:** Characteristics of the patients *N* = 48

Characteristics		Number	Percentage
Sex	Male	29	60
	Female	19	40
Age	0–44	4	8
	45–59	7	15
	60–74	15	31
	75–90	20	42
	>90	2	4
Main Diagnosis	Cancer	39	82
	Neurodegenerativ Diseases	5	10
	Organ Failure	4	8
Advanced Directive	Yes	0	0
	No	52	100

The reasons for presentations were: generalized weakness 21.5 %, social isolation 13.7 % and exams realization 9.8 % (meaning transfers to the ED motivated solely by a wish to be examined, with the goal to return home after). Other reasons were: dyspnea, end of life and pain (7.8 % each), respiratory distress and intervention impossible at home (5.9 % each), occlusion and bleeding (3.9 % each). Other reasons were negligible in number.

Diagnoses made by emergency doctors were: generalized weakness and social isolation (15.7 % each), end-of-life support (13.7 %). Other reasons were: exams realization, infections and pain (9.8 % each), dyspnea, medical intervention impossible at home (pleural tap, ascites puncture…), bleeding (3.92 % each). Other reasons were negligible in number.

**Fig. 1:**
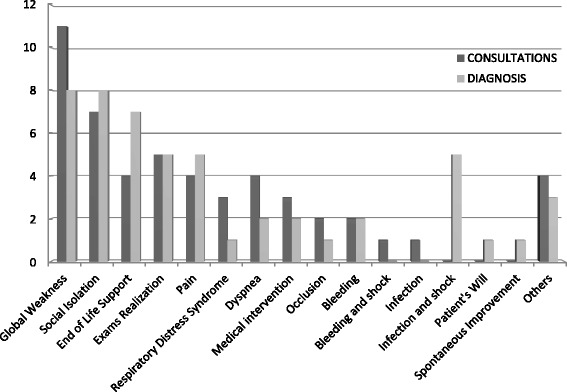
Patient’s reasons for emergency department visits and physicians’ diagnoses, *N* = 52

Reasons for admissions and diagnoses are represented on Fig. 1.

Agreement between patients’ reasons for attending the ED and physicians’ diagnoses was 63 %. It was seen most often for generalized weakness, with eight cases confirmed by emergency doctors. There were some differences regarding infections, social isolation and end of life. Other reasons involved numbers too small to find any agreement or differences.

**Fig. 2:**
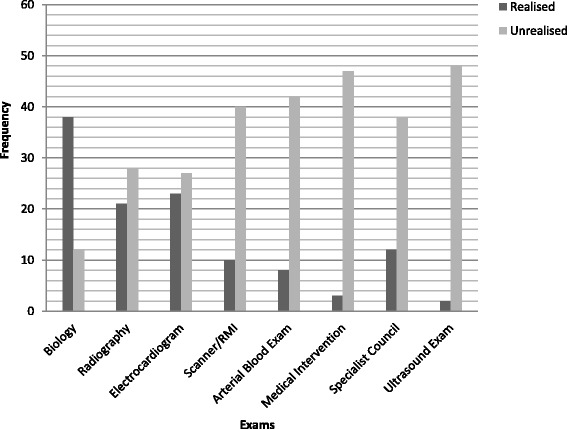
Investigations and treatments performed in emergency departments

Figure 2 shows all the examinations performed during each consultation. Data for two patients were lacking; 56 % of admissions were classified in the avoidable group and 42 % in the unavoidable group; 29 ED transfers were classified as potentially avoidable.

**Fig. 3:**
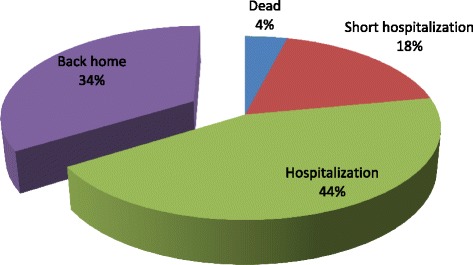
Patient’s outcomes after emegency department visits, *N* = 52

Figure 3 shows patient outcome after the visit. Two files were lacking; 31 patients (62 %) were hospitalized in specialist wards or short post-emergency hospitalization wards, and 17 patients (34 %) returned home. Two patients actually died in the ED.

In 32 % of hospitalizations, specific care was given, and in 68 % patients were hospitalized for observation only.

**Fig. 4:**
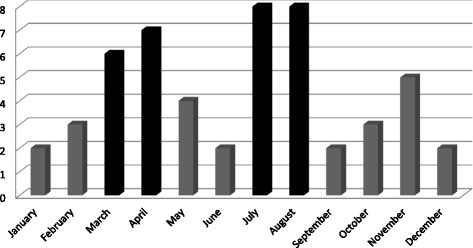
Admission’s repartition in a year

**Fig. 5:**
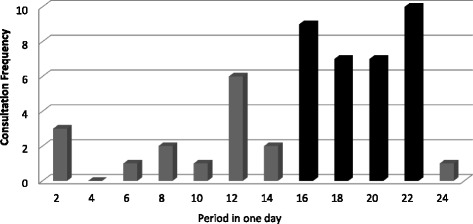
Distribution of the visits on a day

Of presentations, 21 were during holidays, 29 at night and 13 during weekends. The total shows that 44 presentations (85 %) occurred when fewer general practitioners were available (Figs. 4 and 5).

For 29 presentations (56 %), hospital-at-home service was not aware of the emergency problem. HAH was not called before the transport to the ED. We crossed the results for “HAH call” and “avoidable group or unavoidable group”. Potentially avoidable ED presentations were more frequent when patients went directly to the ED without calling the HAH, but this was not statistically significant (*p* = 0,8). When the HAH was not called 18 admissions were potentially avoidable versus 10 when patients called the HAH before presenting to ED.

Patients spent an average of 596 min in the ED, with a broad variability in time; 50 % of them stayed between 120 and 330 min.

Concerning the time to cover the distance between patient’s home and the closest ED : the mean time was 17 min, the longest was 54 and the shortest was 10 min. More than 75 % of patients lived 25 min or less away from the ED.

Focusing on the 4 patients who went to the ED twice, we found no difference between the characteristics of the first and the second presentations or between the other patient’s presentations.

## Discussion

We observed the most frequent reasons/diagnosis for presentations were generalized weakness and social isolation. These are not medical illnesses, but rather descriptions of health or social states. Another French study reached the same conclusion about end of life in emergency departments [14]. Our study is a retrospective one, so we could not evaluate the impact of families’ exhaustion or fear of death at home, which can play an important role in the decision to transfer patients to an ED. Other authors point out the difficulty in evaluating extramedical background [14]. Our results show that the most frequent reasons are not acute medical events, but mostly degradation of chronic complex situations, for which home care is too difficult. Social isolation is not an acute or unpredictable event, the real problem being the degradation of a chronic situation that was initially manageable at home.

These results are strengthened by the number of presentations for end of life (13.7 %). This raises questions because EDs are not designed to deal with end of life [14, 20]. A rise in ED transferral for end of life has also been demonstrated by the French national end-of-life observatory [27].

We observed two reasons for presentation that did not obviously justify ED presentation: pain and examinations. EDs are not designed to perform examinations that could be done safely in other wards. This adds to the possible saturation of ED services so that ED health care professionals cannot perform their roles in due time. Patients have to wait on stretchers in uncomfortable positions with possible complications such as bedsores, fear and disorientation. Pain should be treated by specialized consultations, because it is often due to many factors that evolve in time and need systematic evaluation. Emergency doctors rarely have the whole file and have too little time to explore this symptom from the start. A pain specialist would obtain the best results, but the time to obtain an appointment with a specialist may be long.

A close look at examinations underlined that many of these did not justify ED presentation; 63 % of diagnoses had already been made before the presentation. This situation raises questions because one goal of palliative care is to avoid investigations that are disproportionate and not conducive to patient comfort (WHO definition) [5]. If the investigations do not cause any changes in treatments or diagnosis it is pointless to perform them, and they can be responsible for many complications as stated: pain, fear of transport, orientation trouble, bedsores, iatrogenic effects, etc. We observed that 58 % of presentations led to examinations that could easily have been performed outside the hospital.

Patient outcome after the visits confirmed that transfers to ED are potentially avoidable. One third of patients returned home and out of the patients hospitalized, only one third received specific treatments. The others stayed in hospital only for observation. Hence 75 % of patients presenting to an ED were in the same situation after their emergency presentation: they went back home or were hospitalized with no treatment different from that received at home. If ED presentations do not significantly modify care, then their utility must be challenged. Studies outside France have made the same observations on outcome after ED presentation [28].

We cannot precisely determine the number of potentially avoidable presentations, but if we cross all the results reported above we can estimate that some 58–80 % could be considered avoidable. Unfortunately we found no other studies in the French literature that confirm our conclusions. Studies outside France have found results close to ours: 41–68 % of PAPs [14, 18, 22]. However, we found no international criterion of PAP, so the differences between the studies could be explained by differences in methodology.

We can advance some hypotheses to explain the causes of PAP. First we note the average age of patients: 69.5 years. This was similar in other studies [24, 29]. This age is young compared with life expectancy, so doctors and families may be sending people to EDs in the hope of saving them despite their terminal illnesses; it may be more difficult to give up the struggle with young people.

Our second hypothesis is that most of these presentations occurred when fewer general practitioners were available: holidays, weekends, on-call. Presentations were more frequent in July and August, in the late afternoon or at night and during weekends. Patients went to EDs when their general practitioner was absent. Studies outside France frequently mention this problem [29, 30].

We also observed that 58 % of presentations occurred without first calling the HAH service. Patients were already supervised by a medical structure with a round-the-clock phone service. An Italian study showed that 80 % of situations could be solved just by phone explanations or by a simple medical visit at home in a population of patients with terminal cancer [31]. Hence there is probably also a lack of patient and family information and education. They should first call their carer. A solution can be adopted to stay at home, and if the problem cannot be solved at home, another solution can be organized such as hospitalization directly in a medical ward, avoiding the ED. If there is no other solution, the home hospitalization structure can warn hospital practitioners and give them information to help them provide the best care possible. A French study concluded that a lack of coordination leads to inappropriate readmissions [32].

Finally we analysed the distance between a patient’s home and their closest ED. More than 75 % of patients lived 25 min or less away from the ED. Patients will presumably go more readily to an ED when they are relatively close. However, our study population was too small to demonstrate this.

We make some suggestions to improve the French care system to avoid inappropriate ED presentations. First it seems very important that patients should be able to have a round-the-clock medical consultation.

Another improvement would be to inform and educate patients and families. Patients should consult HAH carers when there is a medical problem before going to an ED. If the final decision is to transfer a patient to the ED, practitioners can send patient information, and care can be adapted accordingly.

We must develop the training of our medical staff to help them make appropriate decisions. Palliative culture has to be transmitted among nurses and doctors in every ward and at home.

In the literature we find the term “home nurses” for night care and observation. Taking care of patients during the night could be a good idea, but is very costly and cannot be extended to a large population of patients.

Our findings require caution: some of the retrospective assessment data were lacking, and our sample of patients was quite small. However, our findings show interesting trends.

A potential bias exists because participants were included through HAH declarations: some data could be lacking if ED presentations were not clearly noted in the files. All the files concerned by the period were checked twice to be sure, but the HAH files were not systematically extracted by the study investigator because of non-authorization in one HAH.

Finally, our definition for potentially avoidable presentations may have caused biased classification. Because of the lack of a standard definition in the literature, we developed this one. It is difficult to know whether this might have resulted in an exaggeration or underestimation of the number of potentially avoidable ED presentations by the patients in our sample.

## Conclusions

Causes of avoidable presentations in France are very close to those found in the international literature. Proposals to improve the French system could probably be adapted to other health systems : to have a round-the-clock medical consultation, patients and families education and medical staff training to palliative care.

## Additional file

Additional file 1: Map of the location of Puy de Dome in France. (DOCX 379 kb)

## Abbreviations

EDs: Emergency Departments
HAH: Hospital-at-home
PAP: Potentially avoidable presentations
WHO: World Health Organization
