# Supplementary figures and images for: Reasons for transferral to emergency departments of terminally ill patients - a French descriptive and retrospective study
Source: BMC Palliat Care. 2016 Oct 21;15:87. doi: 10.1186/s12904-016-0155-y (PMC5073929; doi:10.1186/s12904-016-0155-y)

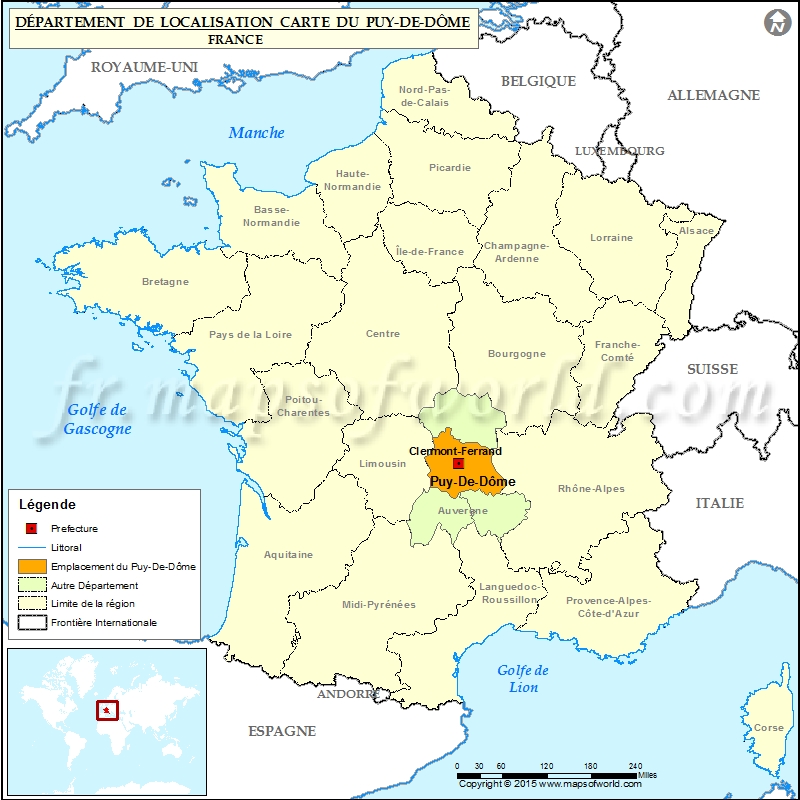

Supplement: Additional file 1: — Map of the location of Puy de Dome in France. (DOCX 379 kb) [file 12904_2016_155_MOESM1_ESM.docx]
